# Inhibition of GATA2 restrains cell proliferation and enhances apoptosis and chemotherapy mediated apoptosis in human GATA2 overexpressing AML cells

**DOI:** 10.1038/s41598-019-48589-0

**Published:** 2019-08-21

**Authors:** Juan Bautista Menendez-Gonzalez, Samantha Sinnadurai, Alex Gibbs, Leigh-anne Thomas, Maria Konstantinou, Alfonso Garcia-Valverde, Magali Boyer, Zhengke Wang, Ashleigh S. Boyd, Allison Blair, Rhys G. Morgan, Neil P. Rodrigues

**Affiliations:** 1 European Cancer Stem Cell Research Institute, Cardiff University, School of Biosciences, Hadyn Ellis Building, Cardiff, CF24 4HQ United Kingdom; 2 Providence Veterans Affairs Medical Center, Alpert Medical School, Brown University, Providence, RI 029208 USA; 3 Department of Surgical Biotechnology, Division of Surgery and Interventional Science, University College London, Royal Free Hospital, London, NW3 2PF United Kingdom; 4 Institute of Immunity and Transplantation, University College London, Royal Free Hospital, London, NW3 2QG United Kingdom; 5 School of Cellular and Molecular Medicine, Biomedical Sciences Building, University of Bristol, Bristol, BS8 1TD United Kingdom; 6 Bristol Institute for Transfusion Sciences, NHS Blood and Transplant, Filton, Bristol, BS34 7QH United Kingdom; 7 School of Life Sciences, University of Sussex, Brighton, BN1 9QG UK

**Keywords:** Acute myeloid leukaemia, Acute myeloid leukaemia

## Abstract

GATA2, a zinc finger transcription factor predominantly expressed in hematopoietic cells, acts as an essential regulator of hematopoietic stem cell generation, survival and functionality. Loss and gain of GATA2 expression has been implicated in myelodysplastic syndrome and acute myeloid leukemia (AML) yet the precise biological impact of GATA2 expression on human AML cell fate decisions remains ambiguous. Herein, we performed large-scale bioinformatics that demonstrated relatively frequent GATA2 overexpression in AML patients as well as select human AML (or AML-like) cell lines. By using shRNAi to target GATA2 in these AML cell lines, and an AML cell line expressing normal levels of GATA2, we found that inhibition of GATA2 caused attenuated cell proliferation and enhanced apoptosis exclusively in AML cell lines that overexpress GATA2. We proceeded to pharmacologically inhibit GATA2 in concert with AML chemotherapeutics and found this augmented cell killing in AML cell lines that overexpress GATA2, but not in an AML cell line expressing normal levels of GATA2. These data indicate that inhibition of GATA2 enhances chemotherapy-mediated apoptosis in human AML cells overexpressing GATA2. Thus, we define novel insights into the oncogenic role of GATA2 in human AML cells and suggest the potential utilization of transient GATA2 therapeutic targeting in AML.

## Introduction

Life-long hematopoiesis is sustained by bone marrow (BM) resident, multi-potent hematopoietic stem cells (HSCs). Extrinsic signals conveyed from the BM niche alongside a cell intrinsic transcriptional program driven by transcription factors (TFs) are crucial for HSC homeostasis and normal hematopoiesis. Chromosomal translocations as well as epigenetic and genetic alterations of TF activity perturb HSC homeostasis leading to the development of acute myeloid leukemia (AML). GATA2, a zinc finger TF predominantly expressed within the hematopoietic system, is an essential, level-dependent regulator of HSC generation, survival and functionality^1,2^. Exemplifying its role as a crucial regulator of HSC behavior, perturbations of GATA2 expression have been observed in AML^3,4^. GATA2 loss of function mutations cause immunodeficiency syndromes that progress to myelodysplastic syndrome and AML on acquisition of secondary mutations^5–7^. Conversely, overexpression of GATA2 has been implicated in the pathogenesis of pediatric and adult AML^4,8^ and low-level overexpression of GATA2 immortalizes murine BM *in vitro* without leading to myeloid neoplasia *in vivo*^9^. As of yet, the precise biological impact of GATA2 overexpression on human AML cell fate decisions remains unclear. In this study, we therefore explore the requirement for GATA2 expression in human AML cells.

## Material and Methods

### AML cell lines and inhibitors

THP1, HL60, K562, and NOMO1 were cultured in RPMI supplemented with 10% FBS. K-7174 (Bioquote limited) and Etoposide (VP16; Sigma) were dissolved in DMSO. Ara-C (Cytosine β-D-arabinofuranoside; Sigma) was dissolved in PBS. Inhibitor assays were performed for 48 hours.

### Colony forming cell (CFC) assay

Colony forming cell (CFC) assay was performed by plating 2,000 THP1 cells into methylcellulose H4434 (STEMCELL technologies) following the manufacturer’s instructions.

### Bioinformatics analysis

AML and control patient datasets were downloaded from GEO^10^ and ArrayExpress^11^ to yield a case/control AML cohort hybridized to the same array (Affymetrix Human Genome U133 Plus 2.0 GeneChip). AML cohort (n = 2611), control cohort: (n = 77) from GEO (GSE14468, GSE22845, GSE10358, GSE12417, GSE13159, GSE14062, GSE15434, GSE16015, GSE38987, GSE22056, GSE33223, GSE17855, GSE15389) and ArrayExpress (E-MTAB-3444). Raw Affymetrix data were downloaded in raw CEL format and imported into an in-house analysis pipeline written in R (version 3.1.1) using Bioconductor^12^ packages from limma^13^, affy^14^ and oligo^15^. Data were normalised using RMA and differentially expressed genes/transcripts were identified using limma “best practice”, and p-values were corrected for multiple testing using Benjamini-Hochberg (false discovery rate). Samples were then run through 2 bespoke R scripts to enable visualization per gene, where the WGNCA package^16^ was used to convert probe-level data to gene-level data. These datasets were then used to produce boxplots.

### Human AML samples

Bone marrow, peripheral blood or leukapheresis samples from patients diagnosed with AML/MDS (Clinical information in Supplementary Table S1) were collected in accordance with the Declaration of Helsinki, with informed consent from Bristol Royal Hospital for Children and Bristol Hematology and Oncology Centre and with approval of University Hospitals Bristol NHS Trust and London Brent Research Ethics Committee. Mononuclear cells were separated using Ficoll-Hypaque (Sigma-Aldrich, Poole, UK) and samples with ≥80% viability included in the study. Normal human BM mononuclear cells purchased from Stem Cell Technologies. Details of samples listed in Supplementary Table S1.

### Generation of lentiviruses

Calcium phosphate method was used to generate lentiviruses^17^. Briefly, lenvitiral vectors containing shRNA against human GATA2 (or scramble control) were diluted in H_2_O, and then mixed with calcium chloride (Sigma). This mix was added drop-wise to 2x hepes buffered saline (HBS; Sigma) and after 15′ incubation, added to the media of HEK293T cells at 70% confluency in a 10-cm dish. Supernatant containing lentiviruses was collected at 48 hours after transfection, passed through a 0.45 μm filter (Sigma), snap freezed in dry ice, and stored at minus 80 °C.

### GATA2 knockdown

Lentiviruses encoding shGATA2 (31–34) (Genecopoeia) linked to GFP or a GFP control vector were bound to the retronectin-coated wells and AML cells were bound to the virus-retronectin-coated plate by centrifugation. Eight hours later, AML cells were transferred to another plate and expanded for 5 days before sorting GFP^+^ cells.

### RNA extraction and gene expression analysis

RNA extraction was carried out with the RNAeasy Plus Micro Kit (Qiagen) according to the manufacturer’s instructions. cDNA was made using the QuantiTec RT Kit (Qiagen) according to the manufacturer’s instructions. Real time quantitative PCR (RT-qPCR) was performed in a QuantStudio® 7 Flex Real-Time PCR System (Applied biosystems) using Taqman (Applied Biosystems) method. Differences in input cDNA were normalised against the housekeeping gene GAPDH (Hs02758991_g1) and the mRNA expression levels of GATA2 (Hs00231119_m1) were determined by the 2^−ΔΔCT^ method of relative quantification^18^.

### Western blot

10 μg of protein extracts were separated in a SDS-PAGE gel, and transferred to PVDF membranes overnight. Membranes were incubated overnight with anti-human GATA2 (clone 3C10.1, Merck Milipore) and detected by a HRP-conjugated anti-mouse IgG secondary antibody and enhanced chemiluminescent (ECL) reagents (WBLUF0100, Merck Millipore). Beta-actin or GAPDH was used as a loading control.

### Flow cytometry

To measure apoptosis, Annexin V assay (BioLegend) was performed as described previously^19^. To assess the cell cycle status, AML cells were stained with DAPI (5 μg/mL) and 0.1% NP40 (Sigma). Live cells were enumerated by staining with propidium iodide (20 μg/mL) (Sigma). Data were analysed using FlowJo 10.0.8 (Tree Star, Inc) software and graphed using GraphPad Prism 7 (GraphPad Software Inc, CA). For intracellular flow cytometry, AML cells were fixed with 1% PFA (Thermofisher) for 12′ on ice. Cells were permeabilised with 2% BSA (Thermofisher) and 0.1% Triton-X (Sigma) for 20′ on ice, washed, and stained for 30′ on ice in the dark with anti-human GATA2-PE (IC2046P) (R&D systems). Cells were then washed twice with 2% BSA PBS and analysed in a BD LSR Fortessa IV (BD).

### Statistical analysis

Data presented as mean ± SEM. Significant differences were calculated using Mann–Whitney U test or ONE-WAY ANOVA.

## Results and Discussion

**Figure 1:**
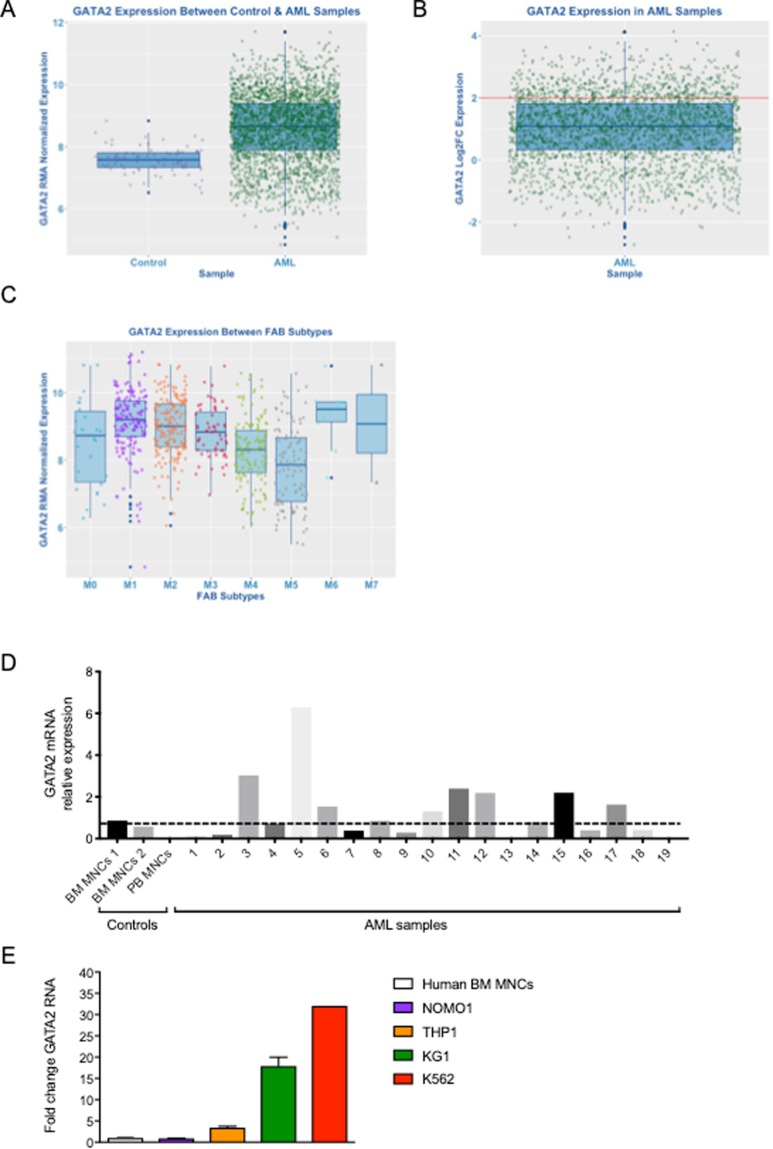
GATA2 is overexpressed in AML. (**A**) Bioinformatics analysis of GATA2 expression between control and AML patients. (**B**) GATA2 expression in AML samples. Red line marks GATA2 overexpressing samples (25%) based on Log2FC. (**C**) GATA2 expression between FAB subtypes in AML patients (**D**) GATA2 RNA levels from human AML patients. Dotted line marks GATA2 over-expressing samples in comparison to healthy BM control samples. (**E**) RNA from NOMO1, THP1, KG1a and K562 was extracted and GATA2 levels assessed by qPCR. Human BM MNCs were used as a control. GAPDH was used as a housekeeping gene. n = 2–5 from 2 independent experiments.

We initially sought to uniformly evaluate GATA2 expression in a large cohort of AML patients. AML (n = 2611) and control (n = 77) patient datasets were downloaded from Gene Expression Omnibus (GEO) to create a case/control cohort hybridised to the same array (Affymetrix Human Genome U133 Plus 2.0 GeneChip) and analyzed through R using bio-conductor packages, where data was normalized using Robust Multi-array Average (RMA). We found that GATA2 expression was higher in AML patients compared to healthy controls (BM MNCs) and was overexpressed in 25% of AML samples (Fig. 1A,B). GATA2 overexpression was observed across FAB-subtypes in AML patients, including M1, M2, M3 and M6 subtypes while M5 AML expressed normal GATA2 level (Fig. 1C). Mirroring this data, we detected GATA2 overexpression in 8 out of 19 samples (42%) in primary AML patient samples (Fig. 1D). When assessing GATA2 gene expression in human AML (or AML-like) cell lines, we observed that, compared to normal BM MNCs, GATA2 expression was increased by 20-fold in KG1 cells and 30-fold in K562 cells (Fig. 1E). We also gauged GATA2 gene expression in two AML cell lines harboring the MLL-AF9 translocation; THP1 cells showed a 4-fold increase in GATA2, while NOMO1 cells had similar GATA2 expression compared to control BM MNCs (Fig. 1E). From this data we infer that GATA2 overexpression is relatively prevalent in human AML.

**Figure 2:**
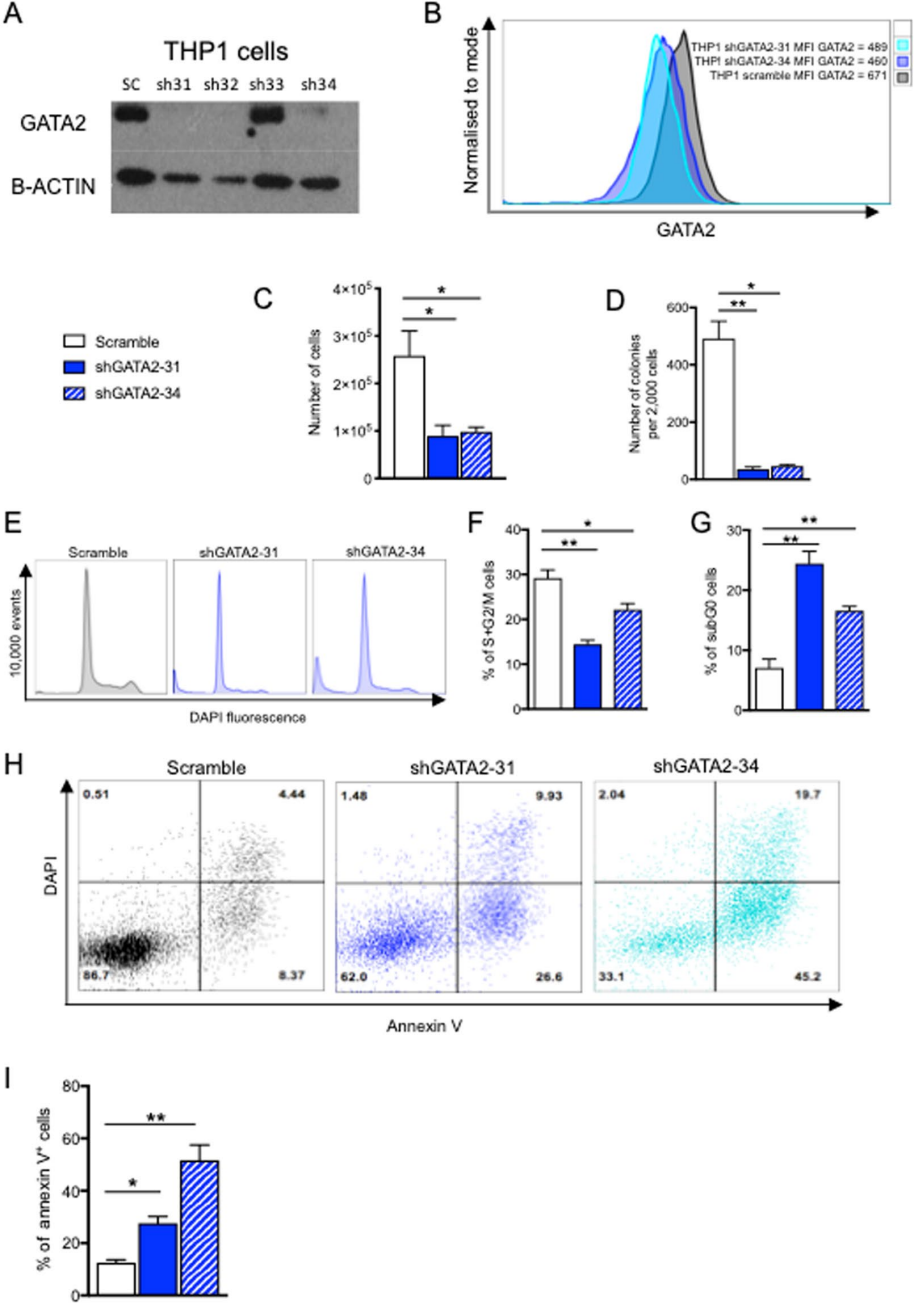
GATA2 knockdown impedes cell proliferation and induces apoptosis of THP1 AML cells. THP1 cells were transduced with lentiviruses encoding a short hairpin against human GATA2 (or scramble control) and a GFP reporter. GFP+ cells were FACS-sorted and cultured for 72 hours. (**A**) Western blot showing GATA2 knockdown in THP1 cells. Un-cropped Western blot shown in Supplementary Fig. 1 (**B**) Flow cytometry histogram showing GATA2 protein levels in shGATA2 31 and 34 compared to control using intracellular flow cytometry. (**C**) Number of live cells at 72 hours after sorting (n = 4). (**D**) Number of CFCs at day 12 (n = 4). (**E**) Flow cytometry histogram showing cell cycle profile at 72 hours after sorting (**F**,**G**). Frequency at 72 hours after sorting of (**F**) proliferating cells (S + G2/M) and (**G**) apoptotic cells (subG0/G1) (n = 4). (**H**) FACS plots showing increased apoptosis after GATA2 knockdown 72 hours after sorting. (**I**) Frequency of apoptotic cells (Annexin V+) at 72 hours after sorting (n = 4). Data are mean ± SEM. Statistical analysis: Mann–Whitney U test.

**Figure 3:**
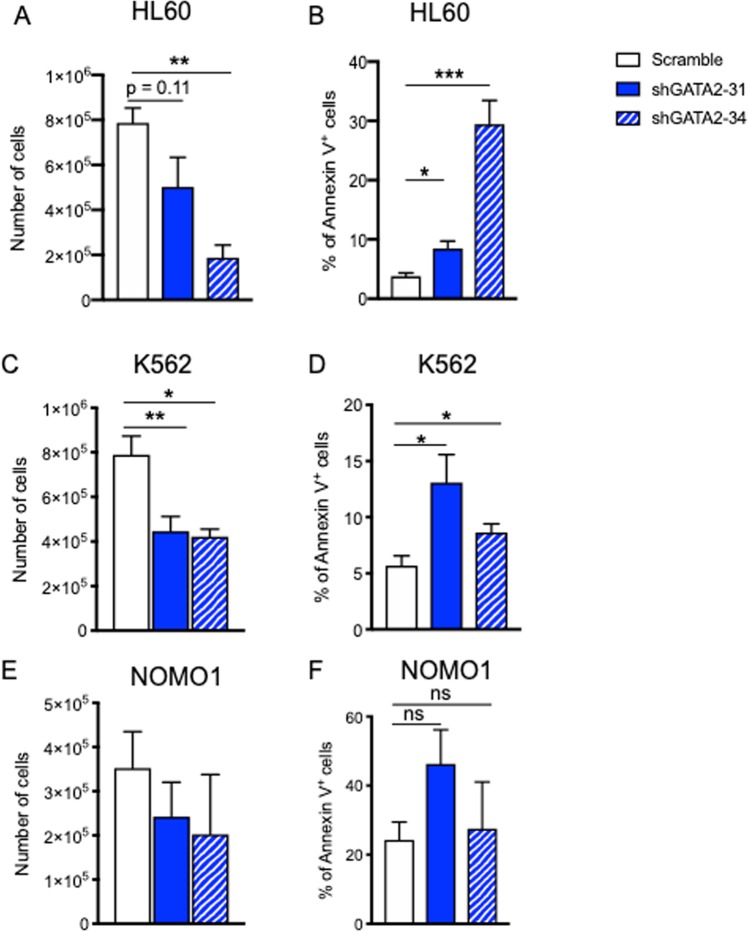
GATA2 knockdown impairs leukemia growth/survival of HL60 and K562 cells but does not affect NOMO1 cells. HL60, K562 and NOMO1 cells were transduced with lentiviruses encoding a short hairpin against human GATA2 (or scramble control) and a GFP reporter. GFP+ cells were FAC-sorted and cultured for 72 hours. (**A**) Number of live HL60 cells 72 hours after sorting (n = 4). (**B**) Frequency of apoptotic HL60 cells (annexin V+) 72 hours after sorting (n = 4). (**C**) Number of live K562 cells 72 hours after sorting (n = 3–4). (**D**) Frequency of apoptotic K562 cells (annexin V+) 72 hours after sorting (n = 4). (**E**) Number of live NOMO1 cells 72 hours after sorting (n = 3–4). (**F**) Frequency of apoptotic NOMO1 cells (annexin V+) 72 hours after sorting (n = 4). Data are mean ± SEM. Statistical analysis: Mann-Whitney test.

Yet the precise biological impact of GATA2 overexpression on human AML cell fate decisions remains unclear. To address this, we employed a bicistronic lentiviral vector system carrying a GFP reporter and shRNA targeted to GATA2 expression in THP1 cells. Efficient knockdown of GATA2 protein (Fig. 2A,B, Supplementary Fig. 1) and RNA (Supplementary Fig. 2) was confirmed in three out of four knockdown (KD) constructs. Proliferation and clonal (CFC) growth capacity of GATA2 KD THP1 cells was compromised compared to control THP1 cells (Fig. 2C,D). When cell cycle status of GATA2 KD THP1 cells was evaluated, we found a reduction in the frequency of proliferating cells (S + G2/M) (Fig. 2E,F). The cell cycle profile of GATA2 KD THP1 cells also revealed an increase in the subG0/G1 population, indicative of diminished cell survival (Fig. 2E,G) and, congruent with this observation, the Annexin V assay confirmed that GATA2 KD induced apoptosis of THP1 cells (Fig. 2H,I). To validate that these effects were not THP1 cell-specific, we repeated these experiments in another AML line, HL60, and a myeloid leukemia cell line, K562 (derived from a chronic myeloid leukemia patient in blast crisis and therefore akin to AML); both these cell lines overexpress GATA2 (Fig. 1 and ^8^). In agreement with results in GATA2 KD THP1 cells, we observed an impact on cell proliferation and survival in HL60 and K562 cells following GATA2 KD (Fig. 3A–D, Supplementary Figs 2, 3). This phenotype was not replicated in NOMO1 cells (Fig. 3E,F, Supplementary Fig. 2), where GATA2 was expressed at a normal level. These data collectively demonstrate that inhibition of GATA2 expression forestalls AML cell proliferation and survival in the setting of GATA2 overexpression, and suggest the potential for AML therapeutic targeting in this context.

**Figure 4:**
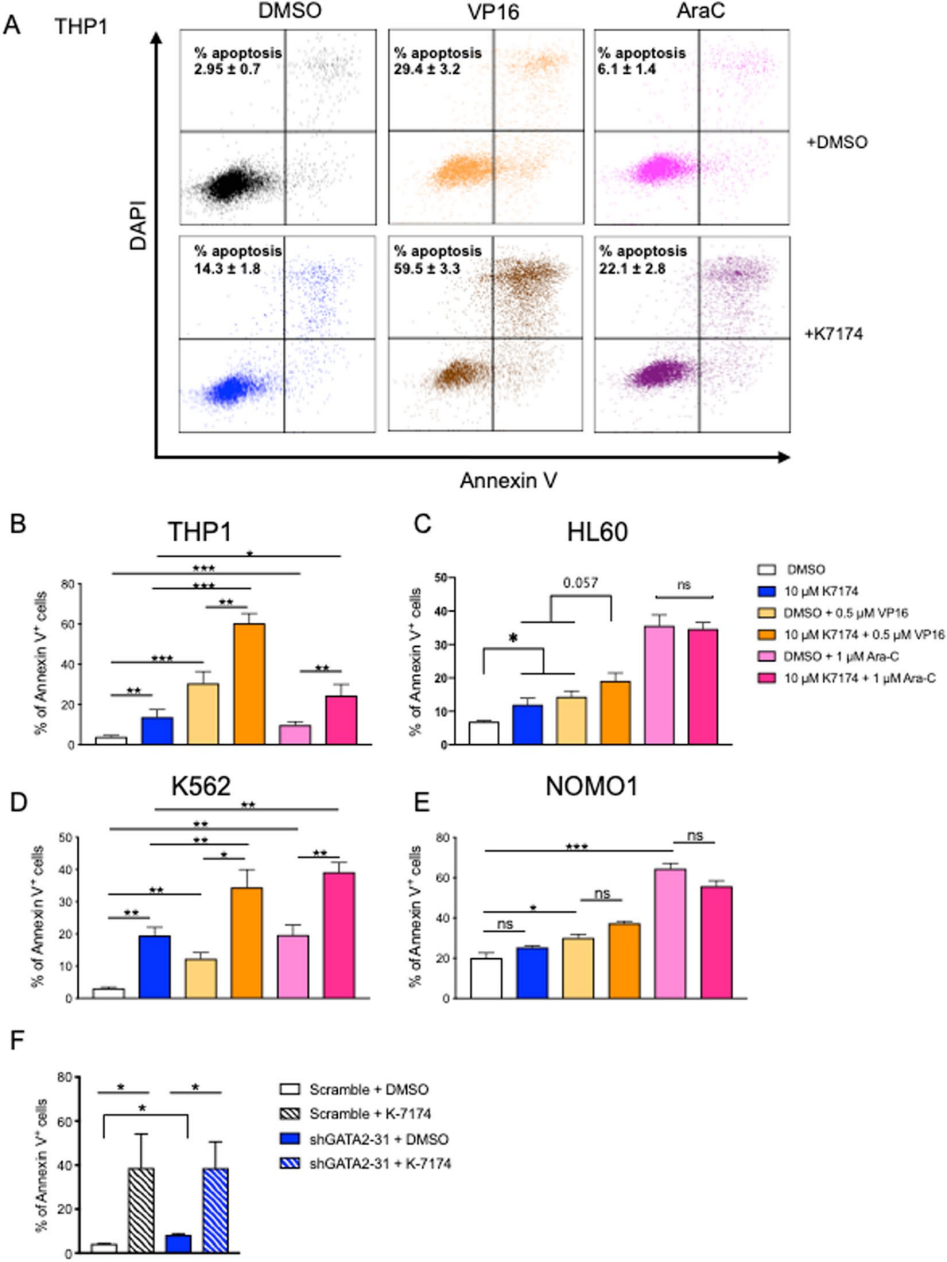
Short-term pharmacological inhibition of GATA2 enhances the killing activity of AML chemotherapeutics. THP1, HL60, K562, and NOMO1 cells were treated with K-7174 alone or in combination with VP16 or Ara-C for 48 hours. (**A**) Representative flow cytometric plots showing increased apoptosis after K-7174 treatment alone or in combination with chemotherapeutics in THP1 cells. (**B**–**E**) Bar graph showing levels of annexin V^+^ cells in (**B**) THP1, (**C**) HL60, (**D**) K562, and (**E**) NOMO1 cells at 48-hour time point (n = 4). (**F**) Bar graph showing levels of annexin V^+^ cells in scramble and shGATA2-31 KD THP1 cells treated with K-7174 for 72 hours (n = 3–4). Data are mean ± SEM. Statistical analysis: ONE-WAY ANOVA.

Using a combination of specific small molecule inhibitors with standard AML chemotherapeutics is a desirable treatment option, especially if this enables a calibrated reduction of standard chemotherapeutics and its related toxicity. We asked whether pharmacological GATA2 inhibition could improve the killing activity of the AML chemotherapeutics Cytarabine (Ara-C) and Etoposide (VP16). By incubating THP1, HL60 and K562 cells with K-7174, a pharmacological inhibitor of GATA2^20–22^, either alone or in combination with VP16 or Ara-C for 48 hours, we found that K-7174 treatment augmented the number of apoptotic THP1 and K562 cells and that addition of K-7174 to VP16 or Ara-C increased its ability to eradicate THP1 and K562 cells (Fig. 4A,B,D). HL60 cells were susceptible to K7174 treatment alone or in combination with VP16, but not with Ara-C (Fig. 4C). In contrast, K-7174 used alone or in combination with VP16 or Ara-C had no impact on the killing activity in NOMO1 cells (Fig. 4E). While knockdown of GATA2 with shGATA2 prior to K7174 treatment reduced relative K7174 mediated killing in THP1 cells (GATA2 KD + K7174: 4.7 fold apoptosis versus K7174 alone: 10.1 fold apoptosis) (Fig. 4F), complete desensitization of THP1 cells to K7174 mediated killing in this setting was not achieved, suggesting that K7174 may have other biological targets. This caveat notwithstanding, overall, direct pharmacological inhibition of GATA2 in GATA2 overexpressing AML cells enhances the killing activity of standard AML chemotherapeutics.

In this report, we identified that GATA2 mediates regulation of cell proliferation and survival in human AML cells overexpressing GATA2. This inference is consistent with independent observations in the GATA2 overexpressing AML cell line KG-1 (Fig. 1E and ^23^). Yet GATA2 inhibition did not impact NOMO1 cells, which exhibit normal GATA2 expression. It is possible that persistent GATA2 inhibition could alter the proliferative and survival capacity of AML cells with normal GATA2 expression. Notably, the extent of impact of GATA2 knockdown in leukemic cells appears to be independent of basal GATA2 overexpression level (Figs 1E, 2–4 ^8^ and unpublished observations), which may reflect differential dose-dependent transcriptional requirements for GATA2 in myeloid leukemia subtypes or in pediatric versus adult patients. We propose transient, pharmacological inhibition of GATA2, in collaboration with standard chemotherapeutics, as a novel therapeutic strategy in AML cases where GATA2 is overexpressed. That effects were observed in cell lines derived from patients with pediatric AML (THP1) or adult AML (HL60) suggests that inhibiting GATA2 will be clinically effective in both pediatric and adult AMLs that overexpress GATA2. Alternatively, as opposed to directly targeting GATA2, it may feasible to therapeutically target the GATA2 transcriptional network in these AML settings. Given that relative GATA2 expression increases in AML patients after chemotherapy^23^, beneficiaries of this therapy could extend beyond those patients who overexpress GATA2 at diagnosis. GATA2 mediated therapeutic strategies should also focus on human AML leukemic stem cells, where GATA2 expression is up-regulated^24^ and where inducing cell death would be an attractive strategy to eliminate the provenance of leukemic cell growth.

## Supplementary information

Supplementary Information
