# Supplementary material for: Inhibition of GATA2 restrains cell proliferation and enhances apoptosis and chemotherapy mediated apoptosis in human GATA2 overexpressing AML cells
Source: Sci Rep. 2019 Aug 21;9:12212. doi: 10.1038/s41598-019-48589-0 (PMC6704064; doi:10.1038/s41598-019-48589-0)
Supplement: Supplementary file 1 — Supplementary Information [file 41598_2019_48589_MOESM1_ESM.pdf]

SUPPLEMENTARY INFORMATION

Inhibition of GATA2 restrains cell proliferation and enhances apoptosis and chemotherapy mediated apoptosis in human GATA2 overexpressing AML cells

Juan Bautista Menendez-Gonzalez[1], Samantha Sinnadurai[1], Alex Gibbs[1], Leigh-anne Thomas[1], Maria Konstantinou[1], Alfonso Garcia-Valverde[1], Magali Boyer[1], Zhengke Wang[2], Ashleigh S. Boyd[3,4], Allison Blair[5,6], Rhys G. Morgan[5,7] and Neil P. Rodrigues[1]

[1]European Cancer Stem Cell Research Institute, Cardiff University, School of Biosciences, Hadyn Ellis Building, Cardiff CF24 4HQ, United Kingdom

[2]Providence Veterans Affairs Medical Center, Alpert Medical School, Brown University, Providence, RI 029208, USA

[3]Department of Surgical Biotechnology, Division of Surgery and Interventional Science, University College London, Royal Free Hospital, London NW3 2PF, United Kingdom

[4]Institute of Immunity and Transplantation, University College London, Royal Free Hospital, London NW3 2QG, United Kingdom

[5]School of Cellular and Molecular Medicine, Biomedical Sciences Building, University of Bristol, BS8 1TD, United Kingdom

[6]Bristol Institute for Transfusion Sciences, NHS Blood and Transplant, Filton, Bristol BS34 7QH, United Kingdom

[7]School of Life Sciences, University of Sussex, Brighton, BN1 9QG, UK

Corresponding Author:

Neil Rodrigues
Cardiff University, School of Biosciences
European Cancer Stem Cell Research Institute
Maindy Road
CF24 4HQ
United Kingdom

RodriguesN@cardiff.ac.uk

Phone: +44 29206 88507

Supplementary Table S1: Clinical characteristics of AML/MDS patient diagnostic/relapse samples used in this study

ID name	Age	Sex	Clinical details
1	-	-	-
2	10	F	-
3	14	M	APL t(15;17)(q22;q12)
4	7	F	2ry AML/relapse
5	14	F	-
6	10	M	-
7	15	F	MDS/AML
8	11	F	-
9	6	M	2ry AML to Ewing Sarcoma
10	8	F	FAB M4/M5
11	4	M	-
12	-	-	Relapse after BMT, M0/1
13	14	F	MLL rearranged
14	6	M	AML M5
15	6	M	Relapse
16	2	M	t(9;11)(p22;q22), t(11;21)(p23;q8)
17	12	M	AML
18	7	M	t(8;21)(q22;q22)
19	1	M	t(9;11)

Key: APL: acute promyelocytic leukaemia ; MDS: myelodysplastic syndrome
FAB: French-American-British ; BMT: bone marrow transplant
MLL: mixed lineage leukaemia

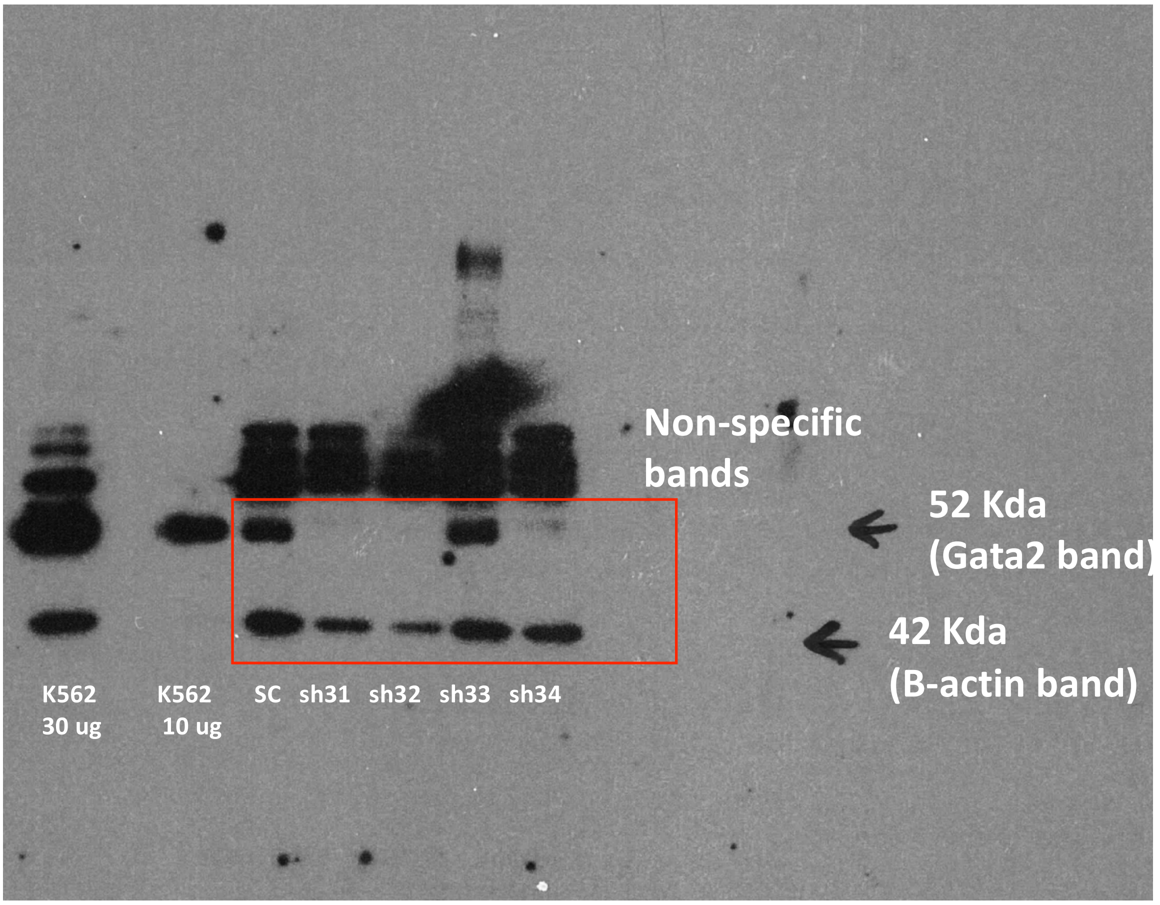

Supplementary Figure 1: Full western blot of figure shown in Figure 2. The red sections indicate results shown in Figure 2A

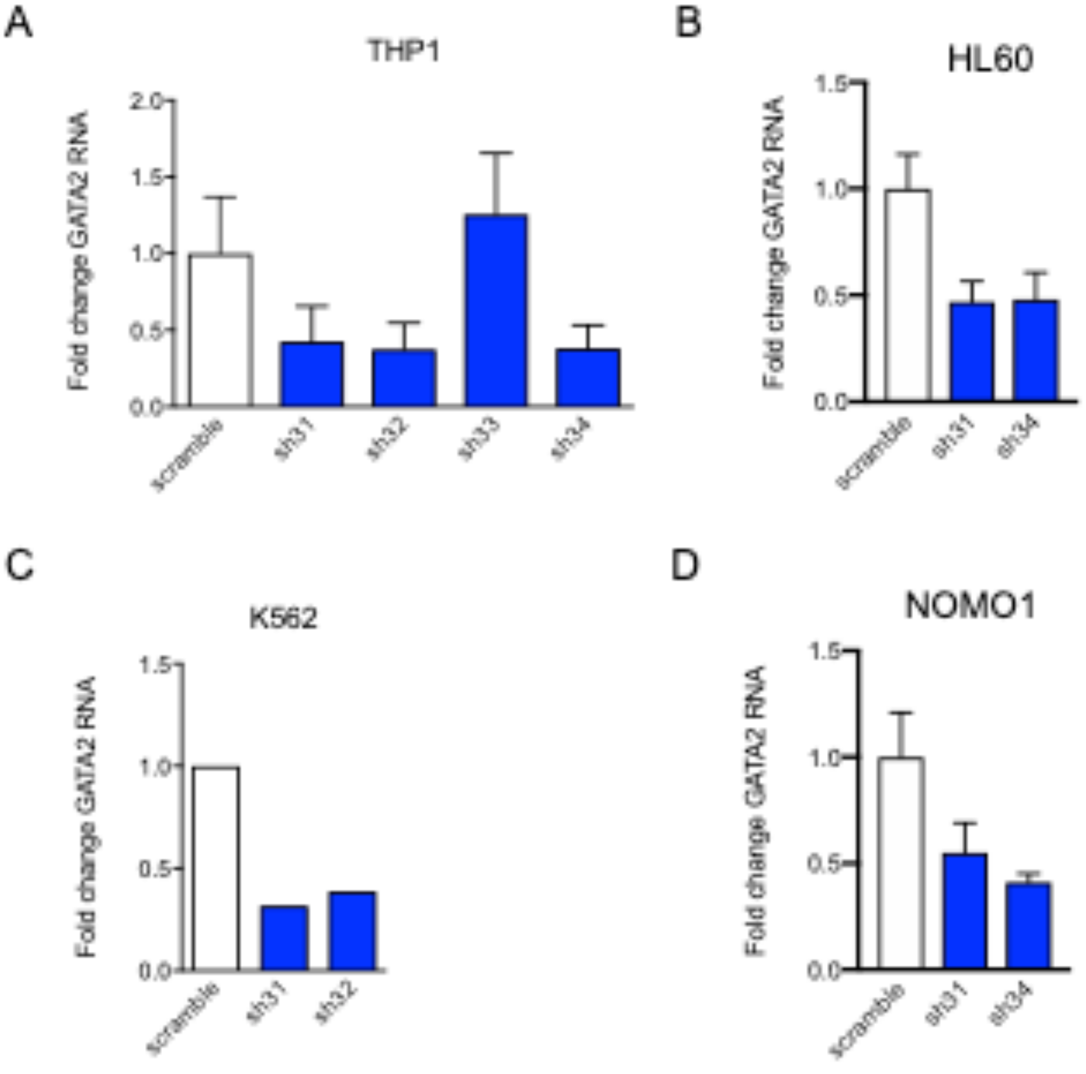

Supplementary Figure 2. shGATA2 validation in AML lines. GATA-2 RNA levels in (A) THP1, (B) HL60, (C) K562 and (D) NOMO1 cells transduced with different shGATA-2 knockdown clones. GAPDH was used as a housekeeping gene. (n=2, except for K562, n=1).

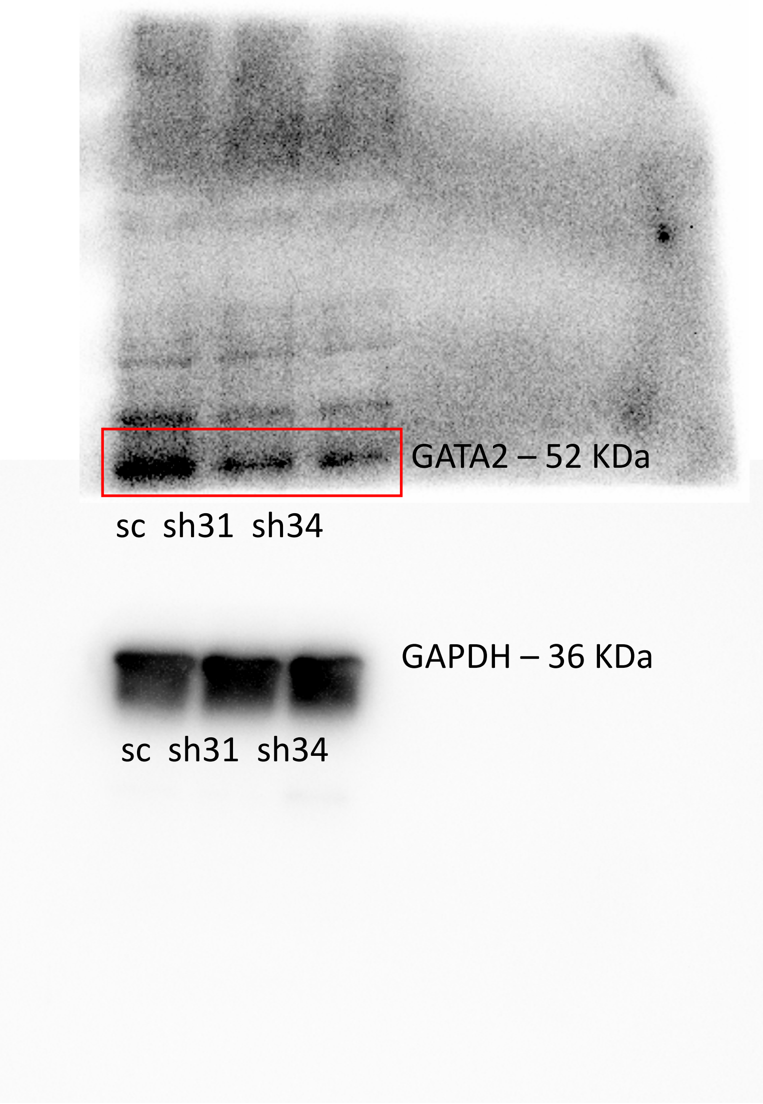

Supplementary Figure 3: Full western blot of HL60 cells after GATA2 KD. The red section indicates the GATA2 protein bands.
